# Pelvic Inflammation

**Published:** 1890-05-10

**Authors:** 


### SURGERY.

### PELVIC INFLAMMATION.

The last number to hand of the *Atlanta Medical Journal* contains a paper on "The Abortive Treatment of Acute Pelvic Inflammations," by Dr. Virgil O'Hardon, Professor of Obstetrics, Atlanta Medical College. The author considers that acute pelvic inflammation is usually associated with septic, gonorrhœal, tubercular, or some other form of inflammation of the tubes. The acceptance of this idea has led to the treatment by removal of the diseased organs (laparotomy). But this is not always practicable. Many patients are unwilling, and many surgeons are unequal to the task. For these reasons an important desideratum is some method by which acute pelvic inflammations may be shorn of their dangerous features without resort to laparotomy. There are two forms of inflammation recognized as occurring in the pelvis, as the result of tubal disease—pelvic cellulitis and pelvic peritonitis. Pelvic cellulitis has a characteristic clinical history, and presents three stages, viz., effusion of serum, solidification, formation of pus. The stage of effusion is of short duration. During this stage examination shows the roof of the passage to possess a boggy œdematous feeling. After about 48 hours the swelling subsides, and the portions of the roof in which it existed are found filled by solid exudate, which is gradually absorbed or becomes purulent. Constitutional symptoms are elevation of temperature, pain in the pelvis extending down the thighs, tenderness of the inguinal regions, painful micturation and defæcation. It is during the stage of effusion that treatment can be successfully applied. This consists in withdrawal of the effusion by the aspirator. The patient is placed across the bed in the dorsal position, with the hips near the edge, and the legs flexed. Three punctures round the os are generally sufficient. The fluid withdrawn is bloody serum, varying in quantity from one to two fluid drachms from each puncture. The relief following the withdrawal of the fluid is immediate. Cases are given of pelvis cellulitis treated by aspiration successfully.

The constitutional symptoms of pelvic peritonitis are very similar to those of pelvic cellulitis. But in the former there is more nausea and prostration. There is an absence of the boggy œdematous feeling so prominent a feature of pelvic cellulitis. Pelvic peritonitis may result in the matting together of all the pelvic viscera, and the end may be disastrous. Hence to abort the disease is a matter of great importance. The author recommends treatment in the early stage by catharsis. As soon as the disease is recognised he gives teaspoonful doses of Epsom salts every hour, until watery stools are voided. From five to eight doses are usually required. When full purging takes place, the pain and temperature begin to decline, and the relief is immediate and marked. The author could cite a number of instances in which he has used this method of treatment successfully.

In connection with the above, we note in the same journal observations by Dr. J. Price, of Philadelphia, on "Pus in the Pelvis; and how to deal with it." The author says there are three methods—electricity, vaginal drainage, and abdominal section. But electricity has no place in the treatment, and vaginal drainage is a crude and inefficient means, and not so safe as some would have us believe. In abdominal sections we have the quickest, correct, most exact, and safest method. A small incision, rapid enucleation of the offending tubes and ovaries, the breaking up and evacuating of the separate pus-pockets, the separation of adhesions, the thorough washing out of the peritoneal cavity by copious irrigation of warm distilled water, the placing of a glass drainage tube in the most dependent portion of the peritoneal cavity, and the careful closing of the abdominal incision, give the patient quick relief, and very often permanent cure.

We do not think Dr. Virgil O. Hardon's treatment will be much adopted, for the simple reason that patients are rarely seen during the first stage of pelvic cellulitis, and because patients are usually averse to operative procedure. And Dr. Price's advice how to get rid of pus in the pelvis will only be acted upon by experts.

